# Chronic hypoxia aggravates monocrotaline-induced pulmonary arterial hypertension: a rodent relevant model to the human severe form of the disease

**DOI:** 10.1186/s12931-017-0533-x

**Published:** 2017-03-14

**Authors:** Florence Coste, Christelle Guibert, Julie Magat, Emma Abell, Fanny Vaillant, Mathilde Dubois, Arnaud Courtois, Philippe Diolez, Bruno Quesson, Roger Marthan, Jean-Pierre Savineau, Bernard Muller, Véronique Freund-Michel

**Affiliations:** 1 INSERM, Centre de recherche Cardio-Thoracique de Bordeaux U1045, F-33000 Bordeaux, France; 2 Univ. Bordeaux, Centre de recherche Cardio-Thoracique de Bordeaux U1045, F-33000 Bordeaux, France; 3 CHU de Bordeaux, F-33000 Bordeaux, France; 4 IHU Liryc, Electrophysiology and Heart Modeling Institute, fondation Bordeaux Université, F-33600 Pessac, Bordeaux, France

**Keywords:** Animal model, Plexiform-like lesions, Pulmonary arterial hypertension

## Abstract

Pulmonary arterial hypertension (PAH) is a severe form of pulmonary hypertension that combines multiple alterations of pulmonary arteries, including, in particular, thrombotic and plexiform lesions. Multiple-pathological-insult animal models, developed to more closely mimic this human severe PAH form, often require complex and/or long experimental procedures while not displaying the entire panel of characteristic lesions observed in the human disease. In this study, we further characterized a rat model of severe PAH generated by combining a single injection of monocrotaline with 4 weeks exposure to chronic hypoxia. This model displays increased pulmonary arterial pressure, right heart altered function and remodeling, pulmonary arterial inflammation, hyperresponsiveness and remodeling. In particular, severe pulmonary arteriopathy was observed, with thrombotic, neointimal and plexiform-like lesions similar to those observed in human severe PAH. This model, based on the combination of two conventional procedures, may therefore be valuable to further understand the pathophysiology of severe PAH and identify new potential therapeutic targets in this disease.

## Introduction

Pulmonary hypertension (PH) is a severe disease characterized by sustained elevated mean pulmonary arterial pressure (mPAP) over 25 mmHg, development of right heart hypertrophy, leading to cardiac failure and finally death [1]. Pathobiology of PH includes pulmonary arterial inflammation, remodeling and altered reactivity, all contributing to increased pulmonary vascular resistances [2].

In the current PH classification, five groups have been identified based on pathophysiological and clinical considerations [1]. The pulmonary arterial hypertension (PAH) group (Group 1) includes idiopathic or familial forms of PH, as well as forms associated to other diseases such as connective tissue diseases or HIV infection [1, 3]. PAH is usually a severe form of PH that combines multiple alterations of pulmonary arteries, including thrombotic lesions, and/or complex and disorganized lesions characterized by a network of proliferated channels separated by core cells, the so-called plexiform lesions [4]. Current pharmacological treatments of PAH manage to slow the progression of the disease but do not afford a cure [5]. Since human samples are difficult to obtain, pertinent animal models are therefore needed to better understand PAH pathobiology and identify new therapeutic targets. However, classical PAH animal models do not recapitulate the severe pathology of human disease [6], and multiple-pathological-insult models have therefore been developed to mimic more closely the human PAH pathophysiology. For instance, pneumonectomy has been associated to MCT injections, leading to neointimal [7] or plexiform-like lesions [8]. In other severe PAH models, SUGEN (SU5416, a tyrosine-kinase inhibitor of the vascular endothelial growth factor receptor VEGFR-2) has been associated to hypoxia [9, 10] or pneumonectomy [11]. However, these models are time consuming and/or require experimented manipulation for surgical procedure.

Our group has developed an alternative rat model of severe PAH, combining MCT injection to 3 weeks of chronic hypoxia (Hx) [6]. Additional preliminary experiments conducted in the same study on 4 rats suggested the development of plexiform-like lesions when the duration of Hx combined to MCT was increased to 4 weeks [6]. Herein, we aimed to confirm the interest of this latter model based on a combination of two conventional procedures: a single MCT intraperitoneal injection associated to 4 weeks of Hx (MCT + Hx rats).

## Description of the model

All animal studies were made according to European and French directives about vertebrate animals protection use for animal experiments. Agreement was obtained from French authorities (number A33-063-907) and all the protocols used were approved by the local ethics committee (Comité d’éthique regional d’Aquitaine, protocol number: 50110016 A).

Male Wistar rats (250-350 g) were randomly assigned into 5 groups: chronic hypoxia (Hx), monocrotaline (MCT), severe PAH (MCT + Hx) for 3 or 4 weeks, and controls (CTRL). Rats exposed to Hx were placed in a hypoxic hypobaric chamber (380 mmHg) for 28 days (4 weeks). In the MCT group, a single intraperitoneal MCT injection (60 mg/kg, Sigma-Aldrich) was performed at Day 1 and rats were maintained in a normobaric/normoxic environment (room air) for 28 days. Severe PAH was induced by combining a single MCT injection (60 mg/kg) at Day 1 with exposure to Hx from Day 2 to Day 28, as previously described [6, 12]. This combined model was also studied at 1, 2 or 3 weeks of chronic hypoxia. Control rats were injected with MCT vehicle and maintained in a normobaric/normoxic environment (room air) for 28 days. Hypobaric chambers were opened three times a week for animal care and cleaning, and all animals had free access to food and water.

For details about other methods and statistical analysis, *see* the online supplemental methods section.

## Findings

**Fig. 1:**
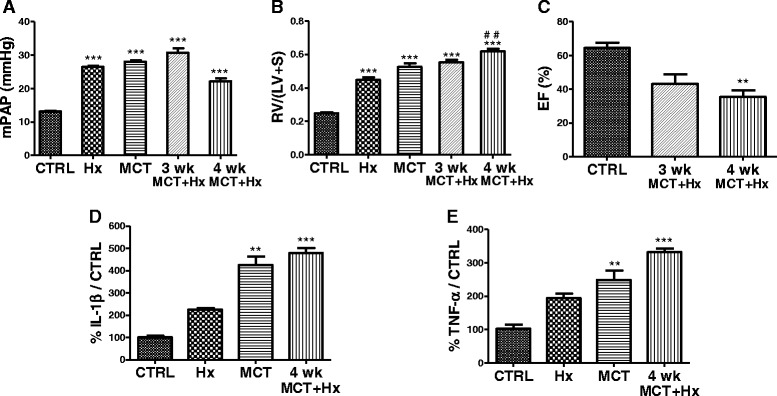
Evaluation of pulmonary arterial hypertension by measuring mean pulmonary arterial pressure, right cardiac remodeling and function, and pulmonary arteries secretion of inflammatory cytokines. **a** Mean pulmonary arterial pressure (mPAP in mmHg) measured in controls rats (CTRL), after 4 weeks of chronic hypoxia (Hx), after 4 weeks of monocrotaline injection (MCT) or after combination of MCT and 3 or 4 weeks of Hx (3wk or 4wk MCT + Hx). Data represent means ± SEM with *n* = 13–14 rats per group. ***p < 0.001 *versus* controls. **b** Right ventricular hypertrophy expressed as the Fulton index (= Ratio of right ventricle weight (RV) to left ventricle plus septum weight (LV + S)) in the same experimental groups. Data represent means ± SEM with *n* = 14–15 rats per group. ***p < 0.001 *versus* controls and ^##^ *p* < 0.01 *versus* Hx. **c** Right heart ejection fraction (EF%) in CTRL or after combination of MCT and 3 or 4 weeks of Hx. Data represent means ± SEM with *n* = 6–8 rats per group. **p < 0.01 *versus* controls. **d**-**e** Secretion of the pro-inflammatory cytokines interleukin-1β (IL-1β, **d**) and tumor necrosis factor-α (TNF-α, **e**) by pulmonary arteries in control rats (CTRL), after chronic hypoxia (Hx), after monocrotaline treatment (MCT), or in rats treated with MCT and exposed to 4 weeks of Hx (﻿4 wk﻿ MCT + Hx). Cytokines were determined by ELISA (results expressed as pg cytokine/ml supernatant after 24 h of incubation in the culture medium and presented as a percentage of cytokine secretion compared to controls). Data represent means ± SEM with *n* = 6 rats per group. **p < 0.01 and ***p < 0.001 *versus* controls. For all experiments, determination of statistically significant differences was assessed with a one-way analysis of variance followed by a Dunn test

By assessing direct mPAP measurements, as previously described [12], we show, for the first time, in MCT + Hx rats, significant increased mPAP values compared to controls after 3 or 4 weeks of our protocol (Fig. 1a). These hemodynamic changes were in accordance with previous studies showing significant increase in right ventricle systolic pressure in this model [6, 13]. Together with increased mPAP values, right ventricular hypertrophy is another hallmark of PH, and we confirm, in the present study a significant right ventricular hypertrophy after MCT + Hx treatment, as previously reported by our group and others [6, 13] (Fig. 1b). However, our results also show that mPAP values were surprisingly lower after 4 weeks than after 3 weeks of the protocol. As reported in human PAH, compensation for right heart failure may be limited in time, and decompensated right ventricular failure then occurs, characterized by diastolic dysfunction and reduced cardiac output [14, 15]. These mechanisms lead to lower mPAP values, as observed in our experiments after 4 weeks of the protocol. To confirm this hypothesis, experiments have been conducted to investigate the right heart function. Functional magnetic resonance imaging evaluated right heart ejection fraction (EF%), using the Simpson’s rule [16]. Our results showed a significant decrease of EF% after 4 weeks of our protocol compared with control animals (Fig. 1c). Such significant decrease was not observed after 3 weeks. These results were confirmed by another technique (pressure-volume loop analysis) showing values of EF% significantly lower after 4 weeks of our protocol compared with values of EF% at 3 weeks (data not shown). These results suggest that our model combining MCT administration with 4 weeks of Hx may therefore be an interesting model of severe PAH, mimicking an advanced stage of the disease.

Complementary to hemodynamic disturbances, structural and functional alterations of pulmonary arteries in human PAH include inflammation, altered reactivity and intense remodeling with a characteristic arteriopathy including thrombotic, neointimal and plexiform lesions [4, 17]. In the previous model combining MCT administration with 3 weeks of Hx, perivascular inflammatory infiltrates were previously evidenced [6]. We further characterized pulmonary arterial inflammation in the present model by showing increased levels of the pro-inflammatory cytokines interleukin-1β and tumor necrosis factor-α released by pulmonary arteries in the MCT + Hx group compared to control animals (Fig. 1d and e). Increased secretion of such pro-inflammatory cytokines has been previously shown in PAH patients [18], with cytokine levels being predictive of outcome in these patients [19, 20]. Reproducing such increase in this experimental model may therefore be of valuable importance in terms of evaluating PAH severity and outcomes.

**Fig. 2:**
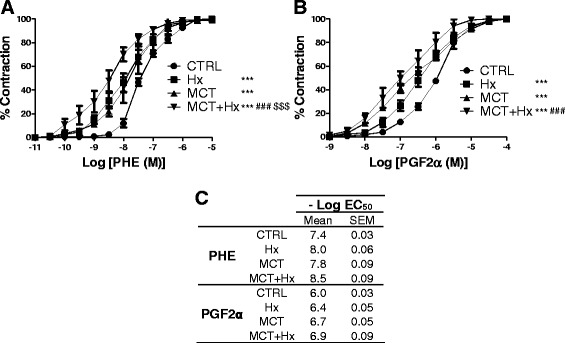
Evaluation of intrapulmonary arterial contraction in pulmonary arterial hypertension models. Contractions of rat intrapulmonary arterial rings induced by cumulative concentrations of phenylephrine (PHE, 10^−11^ to 10^−5^M, **a**), or of prostaglandin F2α (PGF2α, 10^−9^ to 10^−4^M, **b**). Contractions were studied ex vivo after sacrifice at Day 28 in control rats (CTRL), after chronic hypoxia (Hx), after monocrotaline treatment (MCT), of after combination of MCT and 4 weeks of Hx (MCT + Hx). Results are expressed as the percentage of maximal contraction and are presented as means ± SEM with *n* = 12–15 rats per group. Determination of statistically significant differences between concentration-response curves was assessed with a two-way analysis of variance. ***p < 0.001 *versus* control concentration-response curves. ^###^ *p* < 0.001 *versus* Hx concentration-response curves. ^$$$^ *p* < 0.001 *versus* MCT concentration-response curves. Values of -Log EC_50_ (half maximal effective concentrations, means ± SEM) are shown in Table **c**

We also evaluated, for the first time in MCT + Hx rats, pulmonary arterial reactivity by dissecting intrapulmonary arteries from the left lung and mounting them in isolated organ baths, as previously described [12]. Our results show that pulmonary arteries from the MCT + Hx group displayed hyperreactivity to phenylephrine or to prostaglandin F2α (PGF2α), and that this hyperreactivity was even significantly greater compared to that in MCT and/or Hx groups (Fig. 2). In PAH, a resting vasoconstriction of pulmonary arteries contributes to the reduction in vascular caliber [21]. Altered reactivity of pulmonary arteries to vasoconstrictors such as endothelin-1, serotonin, angiotensin II, phenylephrine or PGF2α has been well documented in animal models of PH, in particular those induced by Hx or by MCT [12, 22, 23]. We show, in this study, that the model combining MCT administration to 4 weeks of Hx also reproduces and amplifies this aspect of PAH pathophysiology, and may therefore be helpful to further characterize the pathophysiological mechanisms of pulmonary arterial altered reactivity.

**Fig. 3:**
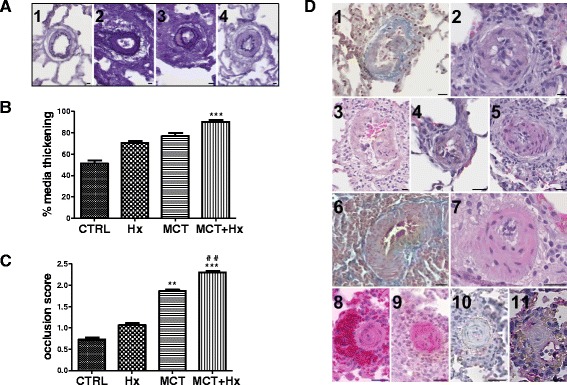
Evaluation of pulmonary arterial remodeling in severe PAH rats. **a**-**c** Remodeling of rat pulmonary arteries (less than 50 μm in diameter) after chronic hypoxia (Hx), after monocrotaline treatment (MCT), or in rats treated with MCT and exposed to 4 weeks of Hx (MCT + Hx), compared to control rats (CTRL). **a** Representative cross-sectional views of remodeled pulmonary microarteries (1: CTRL; 2: Hx; 3: MCT; 4: MCT + Hx) showing medial thickening and luminal occlusion (van Gieson staining). **b** Percentage of medial thickness. **c** Luminal occlusion score. For **b**) and **c**), results are presented as means ± SEM with *n* = 7–11 rats per group. Determination of statistically significant differences was assessed with a one-way analysis of variance followed by a Dunn test. **p < 0.01 and ***p < 0.001 *versus* control. ^##^ *p* < 0.01 *versus* Hx. For **a**) scale bars represent 10 μm. **d** Representative cross-sectional views of lesions observed in small pulmonary arteries of rats treated with MCT and exposed to 4 weeks of Hx. 1–5: pulmonary arterial plexiform-like lesions (stalk-like lesions) (1: picro-Mallory staining; 2–5: hematoxylin and eosin staining). 6: pulmonary arterial thrombotic lesion (Picro-Mallory staining). 7: pulmonary arterial eccentric lesion (hematoxylin and eosin staining). 8–11: concentric cellular neointimal lesions in pulmonary microarteries (8: hematoxylin and eosin staining, 9: α-smooth muscle actin and 10–11: von Willebrand factor immunostainings). Scale bars represent 20 μm

Finally, since there is no curative options in PAH today, many studies currently focus on pulmonary arterial remodeling to define new potential therapeutic targets [17, 24]. In patients with PAH, this remodeling includes pulmonary arterial medial hypertrophy and luminal occlusion, as well as concentric laminar and non-laminar intimal fibrosis, eccentric, plexiform and thrombotic lesions [4, 17]. In the MCT + Hx group, pulmonary arteries displayed classical pathophysiological aspects of PAH, *i.e.* pulmonary arterial medial thickening (Fig. 3a and b) and luminal occlusion (Fig. 3a and c) [6]. We also confirmed the presence of plexiform-like lesions in our model (Fig. 3d 1 to 5), as previously suggested [6]. Interestingly, as described in the Sugen model [9] and by Morimatsu [6], stalk-like plexiform-like complex lesions formed within the blood lumen were observed in the MCT + Hx model (Fig. 3d 1-5). Plexiform lesions observed in human severe PAH are difficult to reproduce in animal models. However, in accordance with our results, some complex lesions, although not reproducing all pathophysiological aspects of human lesions, have been described in other PAH animal models [8, 9], and have also been termed “plexiform-like lesions”. In the complex lesions observed in our model, medial hypertrophy and injured endothelium can be seen. However, the angioproliferative aspect of human plexiform lesions is not reproduced.

We also confirmed the presence of thrombotic lesions (Fig. 3d 6). Thrombotic occlusions have also been observed in other PH models induced by Hx and/or Sugen [25], and are often observed in several forms of human PAH. Although it may be difficult to distinguish between thrombotic lesions and post-mortem coagulation, thrombotic lesions observed in our experiments were observed in PAH rats but not in control animals, suggesting that such thrombosis may rather be caused by the disease itself.

Finally, for the first time in this MCT + Hx model, we also showed the presence of other characteristic human-like lesions. In particular, eccentric lesions were observed (Fig. 3d 7). Although being often difficult to distinguish, such eccentric lesions have also been reported in human PAH and in other PH animal models [26].

In addition, concentric non-laminar intimal thickening lesions were also observed (Fig. 3e 8-11), similar to those observed in human severe PAH [4]. A further characterization of these lesions showed positive staining for α-smooth muscle actin (Fig. 3d 9) but negative staining for the von Willebrand factor (Fig. 3d 10-11). This suggests predominance of smooth muscle cells and/or myofibroblasts rather than endothelial cells in these lesions, in accordance with observations of such lesions in human PAH [4, 27].

If classical characteristics of pulmonary arterial remodeling, *i.e*. medial thickening and luminal occlusion, are easily reproduced in classical models of PAH such as MCT treatment, other severe PAH specific lesions are not observed in these models. This explains the need for developing alternative models more closely related to human PAH pathophysiology. Models such as pneumonectomy associated to MCT injections [7, 8], or the Sugen model [9], lead to lesions very close to the pulmonary arteriopathy observed in human severe PAH. The present model combining MCT and 4 weeks of Hx may be a valuable alternative model, with a protocol realized in a reasonable time and without requiring surgical skills. Nevertheless, this model involves hypoxia chambers that are not available in all laboratories.

In conclusion, we show here that combining MCT injection with 4 weeks of exposure to chronic hypoxia in rats generates a relevant model to the pathogenesis of human severe PAH. In particular, it reproduces multiple structural and functional alterations of pulmonary arteries, including inflammation, altered reactivity and intense remodeling. Moreover, this model displays a pulmonary arteriopathy with thrombotic, severe intimal lesions and some plexiform-like lesions, similar to those observed in human severe PAH. As human samples of PAH are difficult to obtain, the present model, using classical protocols performed in one month, may therefore be valuable to further understand the pathophysiology of severe PAH. According to the current recommendations on PAH translational research suggesting the use of more than one rodent model [28], using this model together with other models of severe PAH may also be of interest to identify new potential therapeutic targets in this disease.

## Abbreviations

CTRL: Control
Hx: Hypoxia
IL-1β: Interleukin -1β
LV: Left ventricle
MCT: Monocrotaline
mPAP: Mean pulmonary arterial pressure
PAH: Pulmonary arterial hypertension
PGF2 α: Prostaglandin F2 α
PH: Pulmonary hypertension
PHE: Phenylephrine
RV: Right ventricle
S: Septum
SEM: Standard error of the mean
TNF-α: Tumor necrosis factor α
